# Attention deficit hyperactivity disorder: genetic association study in a cohort of Spanish children

**DOI:** 10.1186/s12993-015-0084-6

**Published:** 2016-01-08

**Authors:** Clara I. Gomez-Sanchez, Rosa Riveiro-Alvarez, Victor Soto-Insuga, Maria Rodrigo, Pilar Tirado-Requero, Ignacio Mahillo-Fernandez, Francisco Abad-Santos, Juan J. Carballo, Rafael Dal-Ré, Carmen Ayuso

**Affiliations:** 1 Department of Genetics, IIS-Fundación Jiménez Díaz University Hospital (IIS-FJD, UAM), Avda. Reyes Católicos 2, 28040 Madrid, Spain; 2 Centre for Biomedical Research on Rare Diseases (CIBERER), C/Monforte de Lemos 3-5, Pabellón 11, 28029 Madrid, Spain; 3 Department of Pediatrics, IIS-Fundación Jiménez Díaz University Hospital (IIS-FJD, UAM), Avda. Reyes Católicos 2, 28040 Madrid, Spain; 4 Department of Pediatrics, La Paz University Hospital, Paseo de la Castellana 261, 28046 Madrid, Spain; 5 Department of Epidemiology, IIS-Fundación Jiménez Díaz University Hospital (IIS-FJD, UAM), Avda. Reyes Católicos 2, 28040 Madrid, Spain; 6 Clinical Pharmacology Department, Instituto de Investigación Sanitaria Princesa (IP), Hospital Universitario de la Princesa, C/Diego de Leon 62, 28006 Madrid, Spain; 7 Department of Psychiatry, IIS-Fundación Jiménez Díaz University Hospital (IIS-FJD, UAM), Avda. Reyes Católicos 2, 28040 Madrid, Spain; 8 Clinical Research, BUC (Biosciences UAM + CSIC) Program, International Campus of Excellence, Universidad Autónoma de Madrid, Ciudad Universitaria de Cantoblanco, 28049 Madrid, Spain

**Keywords:** Attention deficit hyperactivity disorder, ADHD, Association study, Case–control, *LPHN3*

## Abstract

**Background:**

Attention deficit hyperactivity disorder (ADHD) has a strong genetic component. The study is aimed to test the association of 34 polymorphisms with ADHD symptomatology considering the role of clinical subtypes and sex in a Spanish population.

**Methods:**

A cohort of ADHD 290 patients and 340 controls aged 6–18 years were included in a case–control study, stratified by sex and ADHD subtype. Multivariate logistic regression was used to detect the combined effects of multiple variants.

**Results:**

After correcting for multiple testing, we found several significant associations between the polymorphisms and ADHD (p value corrected ≤0.05): (1) *SLC6A4* and *LPHN3* were associated in the total population; (2) *SLC6A2*, SLC6A3, *SLC6A4* and *LPHN3* were associated in the combined subtype; and (3) *LPHN3* was associated in the male sample. Multivariable logistic regression was used to estimate the influence of these variables for the total sample, combined and inattentive subtype, female and male sample, revealing that these factors contributed to 8.5, 14.6, 2.6, 16.5 and 8.5 % of the variance respectively.

**Conclusions:**

We report evidence of the genetic contribution of common variants to the ADHD phenotype in four genes, with the *LPHN3* gene playing a particularly important role. Future studies should investigate the contribution of genetic variants to the risk of ADHD considering their role in specific sex or subtype, as doing so may produce more predictable and robust models.

**Electronic supplementary material:**

The online version of this article (doi:10.1186/s12993-015-0084-6) contains supplementary material, which is available to authorized users.

## Background

Attention deficit hyperactivity disorder (ADHD) is one of the most common neurodevelopmental disorders in young people, affecting 5.3 % of school-age children [1]. Also, approximately 65 % of children with ADHD continue to show symptoms in adulthood [2].

ADHD is a complex and heterogeneous disorder and its etiology remains unidentified to date [3]. Family, twin and adoption studies have shown that different genes play an important role in the etiology of ADHD, and the mean estimated heritability in childhood is 76 % [4], suggesting that ADHD is one of the psychiatric disorders with the most substantial genetic component.

Many association studies have investigated genetic susceptibility to ADHD. However, efforts to replicate these results have often been poor, yielding inconsistent results as demonstrated in meta-analysis of candidate gene studies [5], but also from linkage studies [6] and genome-wide association studies (GWAS) [7]. ADHD is a complex genetic disorder, in which environmental factors are involved and play a key role [7].

The aim of this study was to test whether previously reported common genetic variants (34 polymorphisms in 18 genes) influence ADHD susceptibility in Spanish patients.

Based on the etiology of ADHD, we chose candidate genes that encode functionally relevant proteins involved in noradrenergic (*SLC6A2, ADRA2A*), dopaminergic (*SLC6A3, DRD2, DRD4, COMT, DDC),* and serotonergic (*SLC6A4, HTR2A, HTR2C*) neurotransmission. In addition, we evaluated other candidate genes frequently reported as being related with ADHD such as *STS*, *FADS2* and *SNAP25*. Finally, significantly reported genes from GWAS studies such as *CDH13, GFOD1, SLC6A9* and *GRM7*, and genes revealed in linkage as playing a role in ADHD susceptibility such as *LPHN3* were included in the study (Table 1).

**Table 1:** Description of the 34 polymorphisms analysed within 18 genes for ADHD

Gene	Description	Variant	Reference
*SLC6A2*	Norepinephrine transporter	rs28386840^a^	[19]
		r5569^c^	[5]
*ADRA2A*	Adrenergic receptor alpha 2A	rs1800544^a^	[5]
		rs553668^e^	[5]
*SLC6A3*	Dopamine transporter	rs2550948^b^	[22]
		rs2652511^b^	[22]
		rs11564750^a^	[22]
		3′UTR VNTR^e^	[5]
		Intron8 VNTR^d^	[5]
*DRD2*	Dopamine receptor D2	rs1800497^f^	[21]
*DRD4*	Dopamine receptor D4	rs3758653^a^	[20]
		Exon3 VNTR^c^	[21]
		Promoter duplication^b^	[21]
*COMT*	Catechol-*O*-methyltransferase	rs4680^c^	[5]
		rs4818^c^	[50]
*DDC*	Dopa decarboxylase	rs6592961^d^	[51]
*SLC6A4*	Serotonin transporter	Promoter VNTR^b^	[5]
		Intron2 VNTR^d^	[5]
*HTR2A*	Serotonin-2A receptor	rs7322347^d^	[51]
*HTR2C*	Serotonin-2C receptor	rs6318^c^	[52]
*SLC9A9*	Glycine transporter	rs9810857^f^	[53]
*GRM7*	Glutamate receptor, metabotropic 7	rs3792452^d^	[54]
*SNAP25*	Synaptosomal-associated protein 25kDA	rs3746544^e^	[5]
*CDH13*	Cadherin 13	rs6565113^d^	[20]
*GFOD1*	Glucose-fructose oxidoreductase domain containing 1	rs552655^d^	[20]
*STS*	Steroid sulfatase	rs12861247^d^	[55]
		rs17268988^d^	[55]
*FADS2*	Fatty acid desaturase 2	rs498793^d^	[31]
*LPHN3*	Latrophilin 3	rs1397548^c^	[17]
		rs2305339^d^	[17]
		rs6551655^d^	[17]
		rs1868790^d^	[24]
		rs6813183^d^	[24]
		rs6858066^d^	[24]

Position in the gene: ^a^upstream gene variant, ^b^ promoter variant, ^c^ exon variant, ^d^ intron variant, ^e^ 3′UTR variant, ^f^ downstream gene variant

## Methods

### Patients and controls

A total of 320 Spanish ADHD patients of Caucasian ancestry and 344 healthy children and adolescents of the same nationality and ancestry were initially included in this case–control study. After a quality control procedure, 290 patients and 340 controls were included in the final analysis. ADHD patients were recruited and evaluated at Fundación Jiménez Díaz University Hospital, whereas the control sample was recruited at both the aforementioned hospital and primary and secondary schools. Exclusion criteria for the control sample included ADHD diagnosis or suspicion of symptomatology, and chronic illness. The sample (cases and controls) comprised subjects between the ages of 6 and 18 years. Even though we did not test for the structure in our cohort, a genome wide study of 800 subjects distributed throughout Spain discarded the presence of genetic stratification [8].

The study protocol was approved by the Research Ethics Committee of the IIS-Fundación Jiménez Díaz University Hospital. The study was conducted according to the tenets of 2008 declaration of Helsinki. Before enrollment, parents or legal guardians signed a written informed consent form after the study objectives and procedures had been explained.

### Clinical assessment

Subjects were included in the study only after a diagnosis of ADHD was made by specialist clinicians according to the diagnostic and statistical manual of mental disorders, fourth edition, text revision (DSM-IV TR) [9]. Each diagnosis was checked by two clinical researchers. Where consensus could not be reached, cases were reviewed by an additional clinical researcher. The children were classified into the following ADHD subtypes: predominantly inattentive subtype, predominantly hyperactive/impulsive subtype and combined subtype.

All cases included underwent clinical assessment using the strengths and difficulties questionnaire (SDQ) for detecting psychological morbidity [10]. Severity of ADHD symptoms was based on the ADHD rating scale-IV (ADHD RS-IV) [11], whereas overall psychosocial functioning was assessed by means of the children’s global assessment scale (CGAS) and the clinical global impression scale (CGI) [12]. Information on obstetric complications, developmental features, medical and psychiatric history, family history, and treatment histories were obtained through maternal interview.

Exclusion criteria included other psychotic disorders (bipolar disorder or schizophrenia among others), pervasive developmental disorders, intelligence quotient (IQ) <70, and neurological damage.

### DNA extraction and genotyping

Genomic DNA samples were obtained either from peripheral blood lymphocytes using an automatic DNA extractor (BioRobot EZ1, Qiagen, Hilden, Germany) or from saliva using the Oragene DNA self-collection kit (DNA Genotek, Kanata, Ontario, Canada), according to the manufacturer’s recommendations. DNA concentration and sample quality were assessed spectrophotometrically (NanoDrop^®^ ND-1000 Spectrophotometer, Wilmington DE, USA).

Candidate polymorphisms were selected based on their relevance as indicated in the literature on ADHD (Table 1).

All single nucleotide polymorphisms (SNPs) were typed using TaqMan Assays-on-Demand or pre-designed SNP genotyping assays following the manufacturer’s instructions (Applied Biosystems, Foster City, CA, USA). PCR and allelic discrimination assays were run using the LightCycler 480 System (Roche Diagnostics, Mannheim, Germany). The results were evaluated using LightCycler^®^ 480 software, version 1.5 (Roche Diagnostics, Mannheim, Germany).

For each variable number tandem repeats (VNTR) polymorphism, subjects were categorized into three genotypes according to the risk allele previously described [5] as follows: *SLC6A3* 3´UTR VNTR (10/10, 10/-, -/-), *SLC6A3* intron8 VNTR (6/6, 6/-, -/-), *DRD4* promoter duplication VNTR (L/L, L/S, S/S), *DRD4* exon3 VNTR (7/7, 7/-, -/-), *SLC6A4* promoter VNTR (L/L, L/S, S/S), *SLC6A4* intron2 VNTR (10/10, 10/-, -/-). Detection of VNTR polymorphisms was performed using fragment analysis. PCR products were visualized on an ABI Prism 3130xl DNA sequencer (Applied Biosystems Foster City, CA). The results were evaluated using the GeneMapper software, version 4.0 (Applied Biosystems, Foster City, CA). Primer sequences and conditions are available upon request.

### Statistical analysis

For the case–control association study, Hardy–Weinberg equilibrium for all genetic variants was assessed only in the control population because deviance from HWE in cases sample might be an indication of association with the disorder; variants not in HWE (p value < 0.01) were excluded from the analysis.

A quality-control procedure was applied to the genotype data. The threshold applied in genotype call rates per sample and per polymorphism was 80 %.

Logistic regression was used to examine the association of the genotype frequencies with the disorder. The effect of the genetic variant on outcome was adjusted by sex and age (covariates). To reduce genetic heterogeneity and to test if there were different genetic factors for the distinct ADHD subtypes, ADHD patients were subdivided into two main diagnostic groups, combined ADHD and inattentive ADHD. The hyperactive-impulsive ADHD subtype was not considered due to its small sample size. To examine differences between males and females, sex-stratified analyses were performed.

Logistic regression analysis was performed to analyze the five inheritance models (codominant, dominant, recessive, overdominant and log-additive) [13] using SNPstats software [14] and expressed as odds ratio (OR), 95 % confidence interval (CI) and nominal significant differences (p value ≤ 0.05). If various inheritance models had significant results, we chose the one with the lowest Akaike information criteria (AIC value).

Genotypes frequencies of variants located on chromosome X (*HTR2C* and *STS* genes) were analyzed only in females.

The Benjamini and Hochberg false discovery rate method was performed for multiple testing corrections [15]. A p value threshold of 0.05 after correction was used to determine significance. Risk-prediction models to investigate the combined impact of multiple genetic variants were applied. For this purpose, polymorphisms with p values ≤ 0.25 were incorporated in a forward stepwise multivariate logistic regression analysis and expressed as the OR, 95 % CI and p value.

The variability explained for each variable as measure of the effect size of the polymorphisms (defined by pseudo-r^2^) and the measure of model predictability (defined by AUC value) were calculated.

A post hoc analysis of statistical power was performed with the CaTS Power Calculator software [16] assuming an OR of 1.5, disorder prevalence of 5 %, significance level of 0.05, and mean minor allele frequency (MAF) observed of 0.30. The statistical power calculated for the final sample included in this study (290 cases and 340 controls) was 89, 64, and 23 % considering an additive, dominant and recessive model, respectively.

## Results

A total of 320 patients and 344 controls were initially investigated. Thirty-four subjects were excluded because they showed genotype call rates <80 %. Therefore, 290 patients and 340 controls were included in the final analysis. Per-marker genotype call rates were higher than 96 % for all variants. The genotype distributions of all polymorphisms were consistent with HWE (p value > 0.01) in the control sample. The average age was 10.43 years (SD 2.95) for ADHD patients and 11.05 years (SD 3.00) for the controls. 80 and 66 % of patients and controls were male, respectively. Clinical classification of the patients was the following: inattentive subtype (n = 102), hyperactive/impulsive subtype (n = 13) and combined subtype (n = 175). Demographic and clinical characteristics of the sample are reported in Table 2.

**Table 2:** Demographic and clinical characteristics of ADHD patients and controls

	ADHD patients	Controls
Age		
Mean (SD)	10.43 (2.9)	11.05 (3)
Range	6–18	6–18
Gender		
Male (%)	230 (79.3)	224 (66)
Female (%)	60 (20.7)	116 (34)
ADHD diagnosis		
Combined type (%)	175 (60.3)	
Inattentive type (%)	102 (35.2)	
Hyperactive type (%)	13 (4.5)	
Previous treatment		
Psychotherapeutic (%)	53 (18.27)	
Pharmacological (%)	22 (7.6)	
Both (%)	52 (17.9)	
No previous treatment (%)	147 (50.6)	
ADHD–RS		
Mean (SD)	27 (12)	
CGI score		
Mean (SD)	3.4 (0.5)	
CGAS score		
Mean (SD)	69 (10)	
Comorbility with (%)		
Learning disabilities	63 (21.7)	
Oppositional defiant disorder	22 (7.6)	
Conduct disorder	16 (5.5)	
Tic disorder	7 (2.4)	

### Logistic regression results for single markers

When the whole sample was considered (unstratified sample), logistic regression analysis for single markers, adjusted by sex and age, showed statistically significant results after correcting for multiple comparisons in two polymorphisms: *SLC6A4* promoter VNTR and *LPHN3* rs2305339 (Table 3; Additional file 1: Table S1).

**Table 3:** Significant results after multiple comparison correction of logistic regression analysis for single markers

Gene	Variant	Model	Genotype	Controls N (%)	Cases N (%)	OR (95 % CI)	p value	p value corrected
All population								
			L/L–L/S	241 (71.1)	239 (83.3)	1		
*SLC6A4*	Promoter VNTR	Recessive	S/S	98 (28.9)	48 (16.7)	0.52 (0.35–0.77)	0.0009	0.0153
			A/A–G/G	177 (52.1)	185 (63.8)	1		
*LPHN3*	rs2305339	Overdominant	A/G	163 (47.9)	105 (36.2)	0.57 (0.41–0.79)	0.0007	0.0153
Combined subtype								
			A/A	174 (51.2)	65 (37.1)	1		
*SLC6A2*	rs28386840	Dominant	A/T–T/T	166 (48.8)	110 (62.9)	1.76 (1.19–2.59)	0.0041	0.0318
			G/G	284 (83.4)	161 (92.5)	1		
			G/C	51 (15.1)	13 (7.5)	0.40 (0.21–0.77)	0.0026	0.0269
*SLC6A3*	rs11564750	Log-additive	C/C	5 (1.5)	0 (0)			
			L/L–L/S	241 (71.1)	151 (87.3)	1		
*SLC6A4*	Promoter VNTR	Recessive	S/S	98 (28.9)	22 (12.7)	0.37 (0.22–0.62)	0.0001	0.0031
			A/A–G/G	177 (52.1)	114 (65.1)	1		
*LPHN3*	rs2305339	Overdominant	A/G	163 (47.9)	61 (34.9)	0.51 (0.34–0.76)	0.0008	0.0124
Male								
			A/A	83 (36.9)	128 (55.2)	1		
*LPHN3*	rs2305339	Codominant	A/G	128 (56.9)	81 (34.9)	0.41 (0.28 –0.60)	0.0000	0.0001

*OR* odds ratio, *CI* confidence interval

p values corrected based on Benjamini and Hochberg method

In the combined ADHD subtype, association was statistically significant for *SLC6A2* rs28386840, *SLC6A3* rs11565750, *SLC6A4* promoter VNTR and *LPHN3* rs2305339. None of the individual comparisons was statistically significant after correcting for multiple comparisons in the inattentive subtype (Table 3 and Additional file 1: Table S1).

In the logistic regression analysis for single markers, adjusted by age, none of the individual comparisons was statistically significant after correcting for multiple comparisons in the female sample. In the male sample, only *LPHN3* rs2305339 remained statistically significant (Table 3 and Additional file 1: Table S1).

### Multivariate logistic regression results

**Fig. 1:**
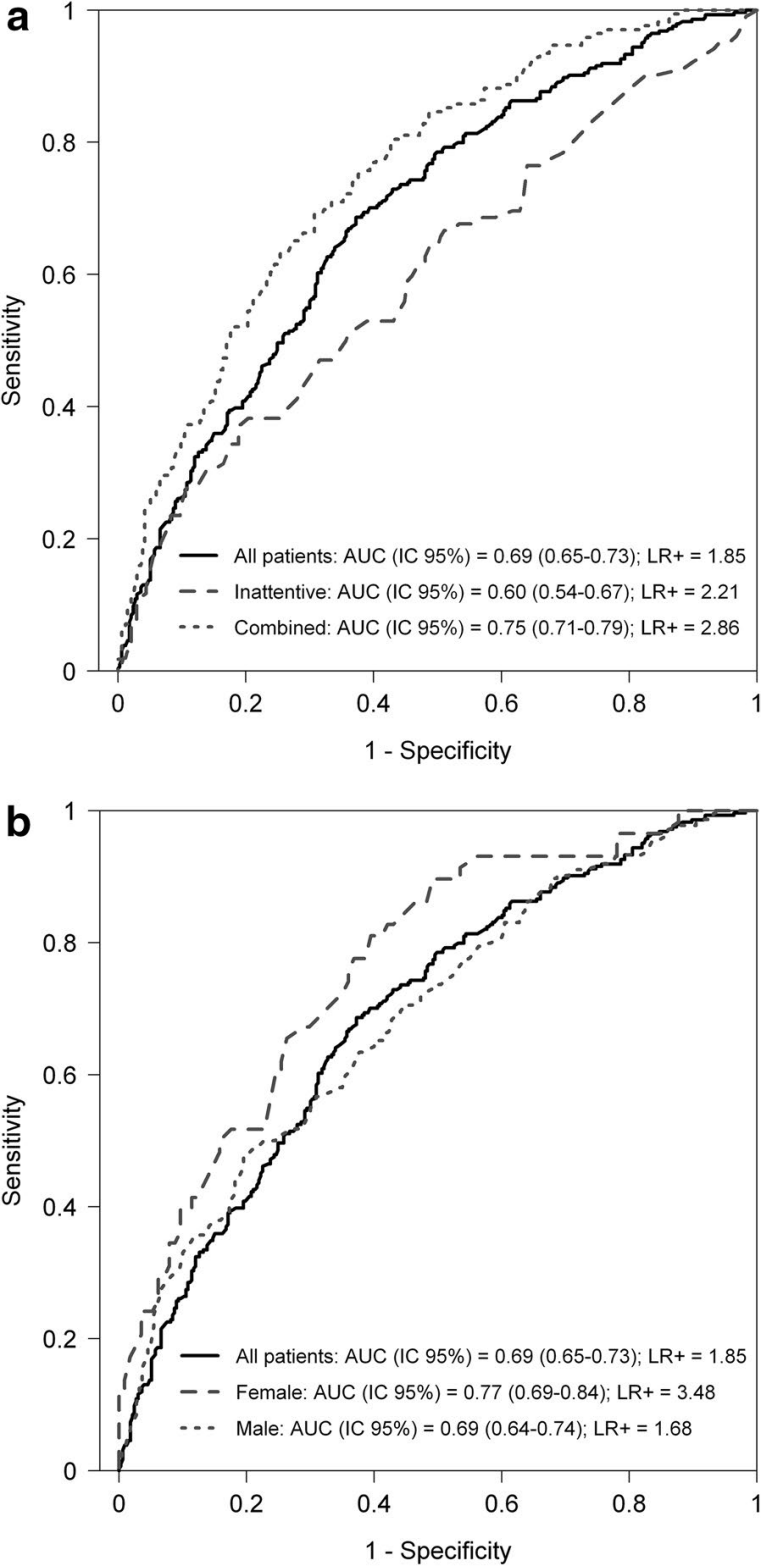
ROC curves analyses of the regression model, stratified by **a** ADHD subtype and **b** sex, and compared to total the population. AUC, area under de curve

**Fig. 2:**
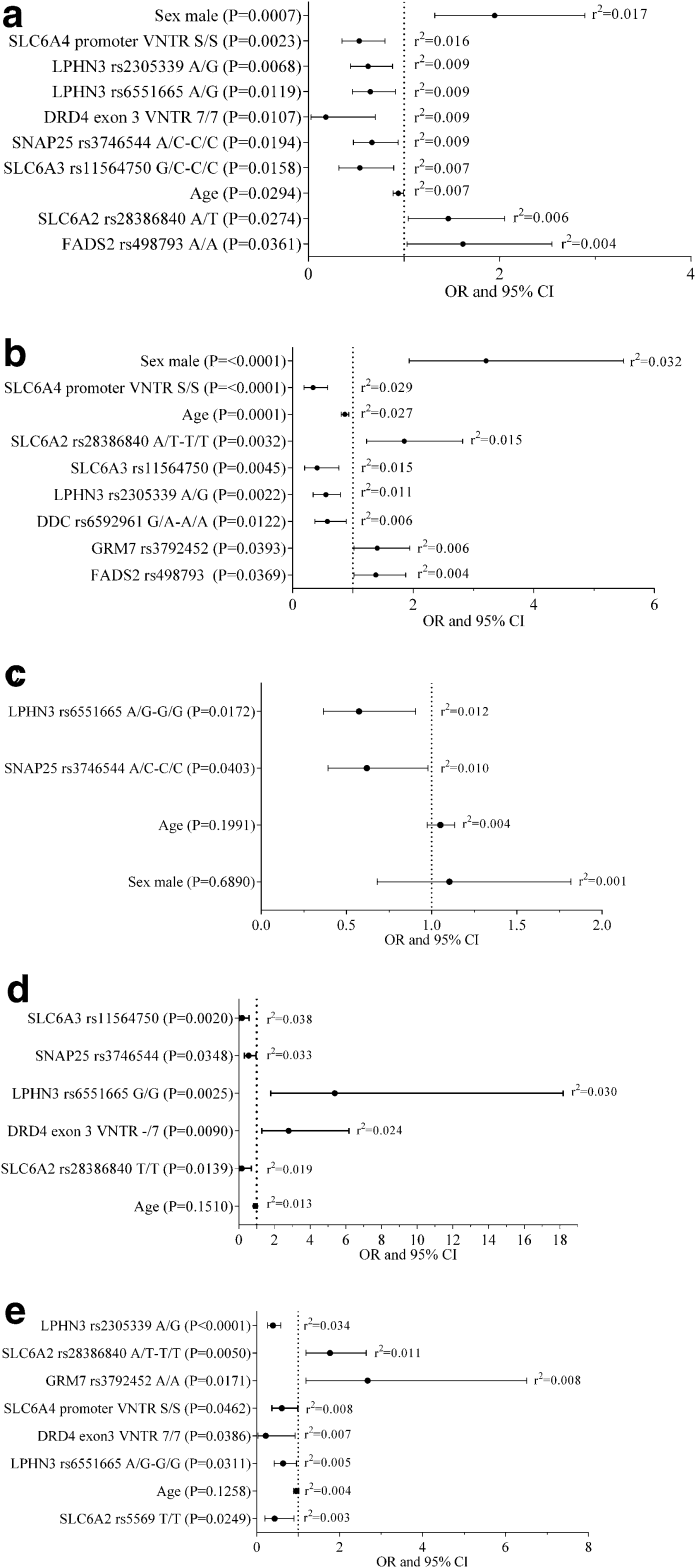
Results for variables that were included in the multivariate regression equation in: **a** the total population, and stratified analyses by **b** combined subtype, **c** inattentive subtype, **d** females and **e** males. The p values, OR 95 % CI, and pseudo r^2^ for the individual variables are shown

Figures 1 and 2 show multivariate logistic regression analyses for the total ADHD sample, subtype and sex stratification. The variables included in the model were ordered according to the amount of variance explained (r^2^).

In the total sample, eight polymorphisms located in seven genes were included in the regression equation: *SLC6A4* promoter VNTR, *LPHN3* (rs2305339, rs6551665), *DRD4* exon3 VNTR, *SNAP25* rs3746544, *SLC6A3* rs11564750, *SLC6A2* rs28386840 and *FADS2* rs498793. The amount of the variance explained for the model was 8.5 % and the AUC was 0.69 (Table 4).

**Table 4:** Overview of multivariate logistic regression analysis

Trait	Pseudo r^2^ (%)	AUC (CI 95 %)	Genetic variants
All sample	8.5	0.69 (0.65–0.73)	8
Combined subtype	14.6	0.75 (0.71–0.79)	7
Inattentive subtype	2.6	0.60 (0.54–0.67)	2
Female	16.5	0.77 (0.66–0.84)	5
Male	8.5	0.69 (0.64–0.74)	7

*AUC* area under de curve, *CI* confidence interval

In the case of the combined subtype, seven polymorphisms located in seven genes were included in the model: *SLC6A4* promoter VNTR, *SLC6A2* rs28386840, *SLC6A3* rs11564750, *LPHN3* rs2305339, *DDC* rs6592961, *GRM7* rs3792453 and *FADS2* rs498793. The amount of the variance explained was 14.6 % and the AUC was 0.75. In the case of inattentive subtype, two polymorphisms were included in the model, *LPHN3* r6551665 and *SNAP25* rs3746544. The amount of the variance explained for the model was 2.6 % and the AUC was 0.60 (Table 4).

Five polymorphisms located in five genes *(SLC6A3* rs11564750, *SNAP25* rs3746544, *LPHN3* rs6551665, *DRD4* exon3 VNTR and *SLC6A2* rs28386840) and seven polymorphisms located in five genes (*LPHN3* (rs2305339, rs6551665), *SLC6A2* (rs28386840, rs5569), *GRM7* rs3792452, SLC6A4 promoter VNTR and *DRD4* exon3 VNTR) were included in the model for females and males, respectively. The amount of the variance explained for the sample including females was 16.5 % and the AUC was 0.77. In the case of males, the amount of the variance explained was 8.5 % and the AUC was 0.69 (Table 4).

## Discussion

This study aimed to both determine whether differential genetic variants may participate in distinct ADHD subtypes and also examine the sex-specific effects of this impact. Multivariate regression analyses of the effects of single genes were evaluated, but as ADHD is a complex polygenic disorder, the combined effect of multiple genes on the phenotype was also considered.

As seen in the logistic regression analysis for single markers, this study provides evidence of a strong association between the *SLC6A4* gene and ADHD in the entire population; and between *SLC6A2*, *SLC6A3* and *SLC6A4* and ADHD in the combined subtype. Special attention should be given to the *LPHN3* gene, since it was associated with the presence of ADHD in the entire population, the combined subtype and the male sample.

In order to clarify the genetic basis of ADHD, the effects of multiple risk factors were examined. In the entire sample, seven genes were included in the regression equation (*SLC6A4, LPHN3, DRD4, SNAP25, SLC6A3, SLC6A2* and *FADS2*). The involvement of these genes in ADHD has been extensively studied [5, 17–23], some in Spanish populations [24, 25]. The contribution of each gene was modest, as expected for a complex genetic disorder (ranging from 0.4 to 1.6 %). The model explained around 9 % of the variance; 7 % of this variance was due to genetic factors. A previous study, including 22 variants, found that 16 % of the variance was due to genetic factors [26].

In the regression equation, two genes were included in the inattentive subtype (*LPHN3* and *SNAP25*) and seven genes (*SLC6A4, SLC6A2, SLC6A3, LPHN3, DDC, GRM7* and *FADS2*) in the combined subtype. A remarkable importance of the *SLC6A4* gene was observed, accounting for 2.9 % of the variance, above the usual threshold of 2 % [27]. This study showed that the clinical subtypes analyzed share genetic risk factors (*LPHN3*), yet S*NAP25* was associated with the inattentive subtype, whereas *SLC6A4*, *SLC6A2, SLC6A3, DDC, GRM7* and *FADS2* were implicated in the combined subtype. The presence of common as well as specific genetic variants for each subtype is supported by previous studies [28, 29]. However, some of these reported associated variants differ between studies [24, 30–36].

The model showed a higher genetic loading for the variables analyzed in combined subtype (14.6 %) than in the inattentive subtype (2.6 %), finding consistent with the previously reported higher genetic loading in ADHD comorbid symptoms [37, 38]. It is important to note the importance of sex and age in the combined subtype (r^2^ 5.9 %) but not in the inattentive sample.

The model for the combined subtype seems to be more predictive than the inattentive subtype (AUC 0.75 and AUC 0.60, respectively) and better than the model used for the whole sample (AUC 0.69). This supports the idea that analyzing more homogeneous phenotypes facilitates the identification of genetic factors.

ADHD is known to have sex-based differences in severity and clinical course [39]. Herein, differences in genetic susceptibility between males and females were observed. In females, *SLC6A3,* *SNAP25, LPHN3, DRD4* and *SLC6A2* genes showed high r^2^ values (range from 2 to 3.8 %). In males, *LPHN3, SLC6A2, GRM7, SLC6A4, DRD4* and *LPHN3* were included in the regression equation. The *LPHN3* gene accounted for 3.4 % of ADHD variability. This analysis showed that genes such as *DRD4, SLC6A2* and *LPHN3* were associated in both sexes, with a stronger effect of SLC6A3 and *SNAP25* in females (r^2^ 3.8 and 3.3 % respectively) and a lesser effect of *GRM7* and *SLC6A4* in males (r^2^ 0.8 %). The association between SLC6A4 and male sample is supported by previous studies [40], but not between SLC6A3 and female sample [41].

To the best of our knowledge, sex-based differences in the genetic risk for ADHD have not been previously reported in the *SNAP25* and *GRM7* genes. These results suggest the need to explore biological evidence of sexually dimorphic effects in such genes.

The percentage of variance explained in females was higher (16.5 %) than in males (8.5 %). The regression model for girls (AUC 0.77) seems to be more predictive than for boys (AUC 0.69). These results suggest that the set of variants analyzed has a higher genetic contribution for ADHD in girls than in boys. Females are less frequently affected because a more extreme genetic load is required for the liability threshold to be surpassed [37, 42]. Additionally, it has been reported that females referred to a clinic are more prone to exhibit other disruptive behaviors [43], although this seems to be a consequence of referral bias [44].

Our results add to extensive literature information about polymorphic variants in genes whose implication in ADHD is widely known through the pathophysiology as *SLC6A2*, *SLC6A*3, *SLC6A4* and *LPHN3*. In some cases the polymorphism associated has a known functional implication, like rs28386840, a functional promoter variant of *SLC6A2* gene. But there are also other polymorphisms associated with any biological meaning that could be in linkage disequilibrium with other unknown functional variants directly involved in genetic susceptibility to ADHD. These findings need to be further explored to improve the understanding of their implication with ADHD.

The conflicting genetic results show the difficulty of replicating across genetic association studies. Often there is important variation in the sample reported, particularly regarding the age, sex ratios and ethnic. Also, an accurate phenotype definition is crucial to obtain successful results in these studies. In our study a rigorously diagnostic criteria was applied.

In addition, the specific effect of a gene could be different depending on the sets of genetic variants analyzed or the model of inheritance evaluated. In contrast with other studies, we evaluated genetic information under different models of inheritance without an a priori consideration of possible genetic effects. This makes it easier to detect genetic effects, since different genotypes of the same gene could be associated with different phenotypes.

The most important limitation of the study was the modest sample size. The statistical power decreased when the sample was subdivided according to ADHD subtype or sex stratification; thus, it is difficult to determine whether negative findings were due to low statistical power or to the absence of a true biological association. On the contrary, we only consider association that remain significant after multiple testing correction in the regression logistic of single markers so we avoid false positive (type I error) rates, giving us confident in the veracity of the results.

## Conclusions

We report evidence of the genetic contribution of common variants to the ADHD phenotype in four genes, with the *LPHN3* gene playing a particularly strong role.

The most predictable model described in this study was for females (r^2^ 16.5 %, AUC 0.77). As seen in this study, analysis of the contribution of multiple genes provides particularly useful insight for the effort to discover the genetic basis of polygenic disorders and multigene analysis had substantial advantages over the single-gene approach. However, the percentage of the variance in ADHD diagnosis explained remains low; hence, most of the genetic component in phenotypic variance remains unexplained when considering common variants. Additional studies including copy number variation [45, 46], exome sequencing studies [47] as well as gene–gene and gene-environment interactions [48, 49] could clarify the genetic contribution to ADHD.

Future studies should investigate the contribution of genetic variants to the risk of ADHD considering their role in specific sex or subtype in order to produce more predictable and robust models, enabling the development of an accurate diagnosis and hopefully improved treatment.

## Additional file

**Additional file 1: Table S1.** Significant results of logistic regression for single markers considering a nominal p value <0.05.

## Abbreviations

ADHD: attention deficit hyperactivity disorder
DSM-IV TR: diagnostic and statistical manual of mental disorders, fourth edition, text revision
SDQ: strengths and difficulties questionnaire
ADHD RS-IV: ADHD rating scale-IV
CGAS: children’s global assessment scale
CGI: clinical global impression scale
SNPs: single nucleotide polymorphisms
VNTRs: variable number tandem repeats polymorphisms
HWE: Hardy–Weinberg equilibrium
CI: confidence interval
AIC: Akaike information criteria
AUC: area under the curve
MAF: minor allele frequency

## References

[1] Polanczyk G, de Lima MS, Horta BL, Biederman J, Rohde LA (2007). The worldwide prevalence of ADHD: a systematic review and metaregression analysis. Am J Psychiatry.

[2] Faraone SV, Biederman J, Mick E (2006). The age-dependent decline of attention deficit hyperactivity disorder: a meta-analysis of follow-up studies. Psychol Med.

[3] Genro JP, Kieling C, Rohde LA, Hutz MH (2010). Attention-deficit/hyperactivity disorder and the dopaminergic hypotheses. Expert Rev Neurother.

[4] Faraone SV, Perlis RH, Doyle AE, Smoller JW, Goralnick JJ, Holmgren MA (2005). Molecular genetics of attention-deficit/hyperactivity disorder. Biol Psychiatry.

[5] Gizer IR, Ficks C, Waldman ID (2009). Candidate gene studies of ADHD: a meta-analytic review. Hum Genet.

[6] Zhou K, Dempfle A, Arcos-Burgos M, Bakker SC, Banaschewski T, Biederman J (2008). Meta-analysis of genome-wide linkage scans of attention deficit hyperactivity disorder. Am J Med Genet B Neuropsychiatr Genet..

[7] Neale BM, Medland SE, Ripke S, Asherson P, Franke B, Lesch KP (2010). Meta-analysis of genome-wide association studies of attention-deficit/hyperactivity disorder. J Am Acad Child Adolesc Psychiatry.

[8] Gayán J, Gallan JJ, González-Pérez A, Sáez ME, Mártinez-Larrad MT, Zabena C (2010). Genetic structure of the Spanish population. BMC Genom.

[9] American Psychiatric Association (2010). Diagnostic and statistical manual of mental disorders.

[10] Goodman R (1997). The Strengths and difficulties questionnaire: a research note. J Child Psychol Psychiatry.

[11] Du Paul G (1991). Parent and teacher ratings of ADHD symptoms: psychometric properties in a community-based sample. J Clin Child Psychol.

[12] Shaffer D, Gould MS, Brasic J, Ambrosini P, Fisher P, Bird H (1983). A children’s global assessment scale (CGAS). Arch Gen Psychiatry.

[13] Iniesta R, Guinó E, Moreno V (2005). Statistical analysis of genetic polymorphisms in epidemiological studies. Gac Sanit.

[14] Sole X, Guino E, Valls J, Iniesta R, Moreno V (2006). SNPStats: a web tool for the analysis of association studies. Bioinformatics.

[15] Benjamini Y, Hochberg Y (1995). Controlling the false discovery rate: a practical and powerful approach to multiple testing. J R Stat Soc Ser B..

[16] Skol AD, Scott LJ, Abecasis GR, Boehnke M (2006). Joint analysis is more efficient than replication-based analysis for two-stage genome-wide association studies. Nat Genet.

[17] Arcos-Burgos M, Muenke M (2010). Toward a better understanding of ADHD: LPHN3 gene variants and the susceptibility to develop ADHD. Atten Defic Hyperact Disord..

[18] Hwang IW, Lim MH, Kwon HJ, Jin HJ (2015). Association of LPHN3 rs6551665 A/G polymorphism with attention deficit and hyperactivity disorder in Korean children. Gene.

[19] Kim CH, Hahn MK, Joung Y, Anderson SL, Steele AH, Mazei-Robinson MS (2006). A polymorphism in the norepinephrine transporter gene alters promoter activity and is associated with attention-deficit hyperactivity disorder. Proc Natl Acad Sci U S A..

[20] Lasky-Su J, Neale BM, Franke B, Anney RJ, Zhou K, Maller JB (2008). Genome-wide association scan of quantitative traits for attention deficit hyperactivity disorder identifies novel associations and confirms candidate gene associations. Am J Med Genet B Neuropsychiatr Genet..

[21] Wu J, Xiao H, Sun H, Zou L, Zhu LQ (2012). Role of dopamine receptors in ADHD: a systematic meta-analysis. Mol Neurobiol.

[22] Genro JP, Polanczyk GV, Zeni C, Oliveira AS, Roman T, Rohde LA (2008). A common haplotype at the dopamine transporter gene 5′ region is associated with attention-deficit/hyperactivity disorder. Am J Med Genet B Neuropsychiatr Genet..

[23] Nyman ES, Ogdie MN, Loukola A, Varilo T, Taanila A, Hurtig T (2007). ADHD candidate gene study in a population-based birth cohort: association with DBH and DRD2. J Am Acad Child Adolesc Psychiatry.

[24] Ribases M, Ramos-Quiroga JA, Sanchez-Mora C, Bosch R, Richarte V, Palomar G (2011). Contribution of LPHN3 to the genetic susceptibility to ADHD in adulthood: a replication study. Genes Brain Behav..

[25] Ribases M, Ramos-Quiroga JA, Hervas A, Sanchez-Mora C, Bosch R, Bielsa A (2012). Candidate system analysis in ADHD: evaluation of nine genes involved in dopaminergic neurotransmission identifies association with DRD1. World J Biol Psychiatry..

[26] Comings DE, Gade-Andavolu R, Gonzalez N, Wu S, Muhleman D, Blake H (2000). Multivariate analysis of associations of 42 genes in ADHD. ODD and conduct disorder. Clin Genet..

[27] Comings DE (1998). Why different rules are required for polygenic inheritance: lessons from studies of the DRD2 gene. Alcohol..

[28] Larsson H, Lichtenstein P, Larsson JO (2006). Genetic contributions to the development of ADHD subtypes from childhood to adolescence. J Am Acad Child Adolesc Psychiatry.

[29] Nadder TS, Silberg JL, Rutter M, Maes HH, Eaves LJ (2001). Comparison of multiple measures of ADHD symptomatology: a multivariate genetic analysis. J Child Psychol Psychiatry.

[30] Mill J, Xu X, Ronald A, Curran S, Price T, Knight J (2005). Quantitative trait locus analysis of candidate gene alleles associated with attention deficit hyperactivity disorder (ADHD) in five genes: DRD4, DAT1, DRD5, SNAP-25, and 5HT1B. Am J Med Genet B Neuropsychiatr Genet..

[31] Brookes K, Xu X, Chen W, Zhou K, Neale B, Lowe N (2006). The analysis of 51 genes in DSM-IV combined type attention deficit hyperactivity disorder: association signals in DRD4, DAT1 and 16 other genes. Mol Psychiatry..

[32] Bidwell LC, Willcutt EG, McQueen MB, DeFries JC, Olson RK, Smith SD (2011). A family based association study of DRD4, DAT1, and 5HTT and continuous traits of attention-deficit hyperactivity disorder. Behav Genet.

[33] Manor I, Eisenberg J, Tyano S, Sever Y, Cohen H, Ebstein RP (2001). Family-based association study of the serotonin transporter promoter region polymorphism (5-HTTLPR) in attention deficit hyperactivity disorder. Am J Med Genet.

[34] Landaas ET, Johansson S, Jacobsen KK, Ribases M, Bosch R, Sanchez-Mora C (2010). An international multicenter association study of the serotonin transporter gene in persistent ADHD. Genes Brain Behav..

[35] Guan L, Wang B, Chen Y, Yang L, Li J, Qian Q (2009). A high-density single-nucleotide polymorphism screen of 23 candidate genes in attention deficit hyperactivity disorder: suggesting multiple susceptibility genes among Chinese Han population. Mol Psychiatry..

[36] Smith TF (2010). Meta-analysis of the heterogeneity in association of DRD4 7-repeat allele and AD/HD: stronger association with AD/HD combined type. Am J Med Genet B Neuropsychiatr Genet..

[37] Hamshere ML, Langley K, Martin J, Agha SS, Stergiakouli E, Anney RJ (2013). High loading of polygenic risk for ADHD in children with comorbid aggression. Am J Psychiatry.

[38] Thapar A, Harrington R, McGuffin P (2001). Examining the comorbidity of ADHD-related behaviours and conduct problems using a twin study design. Br J Psychiatry.

[39] Biederman J, Mick E, Faraone SV, Braaten E, Doyle A, Spencer T (2002). Influence of gender on attention deficit hyperactivity disorder in children referred to a psychiatric clinic. Am J Psychiatry.

[40] Biederman J, Kim JW, Doyle AE, Mick E, Fagerness J, Smoller JW (2008). Sexually dimorphic effects of four genes (COMT, SLC6A2, MAOA, SLC6A4) in genetic associations of ADHD: a preliminary study. Am J Med Genet B Neuropsychiatr Genet..

[41] Das BA, Sarkar K, Ghosh P, Das M, Bhaduri N, Ray A (2013). Significance of dopaminergic gene variants in the male biasness of ADHD. J Atten Disord..

[42] Pauls DL (1991). Genetic factors in the expression of attention-deficit hyperactivity disorder. J Child Adolesc Psychopharmacol..

[43] Gaub M, Carlson CL (1997). Gender differences in ADHD: a meta-analysis and critical review. J Am Acad Child Adolesc Psychiatry.

[44] Ramtekkar UP, Reiersen AM, Todorov AA, Todd RD (2010). Sex and age differences in attention-deficit/hyperactivity disorder symptoms and diagnoses: implications for DSM-V and ICD-11. J Am Acad Child Adolesc Psychiatry.

[45] Williams NM, Zaharieva I, Martin A, Langley K, Mantripragada K, Fossdal R (2010). Rare chromosomal deletions and duplications in attention-deficit hyperactivity disorder: a genome-wide analysis. Lancet.

[46] Ramos-Quiroga JA, Sanchez-Mora C, Casas M, Garcia-Martinez I, Bosch R, Nogueira M (2014). Genome-wide copy number variation analysis in adult attention-deficit and hyperactivity disorder. J Psychiatr Res.

[47] Lionel AC, Crosbie J, Barbosa N, Goodale T, Thiruvahindrapuram B, Rickaby J (2011). Rare copy number variation discovery and cross-disorder comparisons identify risk genes for ADHD. Sci Transl Med..

[48] Ficks CA, Waldman ID (2009). Gene-environment interactions in attention-deficit/hyperactivity disorder. Curr Psychiatry Rep..

[49] Qian Q, Wang Y, Li J, Yang L, Wang B, Zhou R (2007). Evaluation of potential gene-gene interactions for attention deficit hyperactivity disorder in the Han Chinese population. Am J Med Genet B Neuropsychiatr Genet..

[50] Michaelovsky E, Gothelf D, Korostishevsky M, Frisch A, Burg M, Carmel M (2008). Association between a common haplotype in the COMT gene region and psychiatric disorders in individuals with 22q11.2DS. Int J Neuropsychopharmacol.

[51] Ribases M, Ramos-Quiroga JA, Hervas A, Bosch R, Bielsa A, Gastaminza X (2009). Exploration of 19 serotoninergic candidate genes in adults and children with attention-deficit/hyperactivity disorder identifies association for 5HT2A, DDC and MAOB. Mol Psychiatry..

[52] Bobb AJ, Addington AM, Sidransky E, Gornick MC, Lerch JP, Greenstein DK (2005). Support for association between ADHD and two candidate genes: NET1 and DRD1. Am J Med Genet B Neuropsychiatr Genet..

[53] Mick E, Todorov A, Smalley S, Hu X, Loo S, Todd RD (2010). Family-based genome-wide association scan of attention-deficit/hyperactivity disorder. J Am Acad Child Adolesc Psychiatry.

[54] Park S, Jung SW, Kim BN, Cho SC, Shin MS, Kim JW (2013). Association between the GRM7 rs3792452 polymorphism and attention deficit hyperacitiveity disorder in a Korean sample. Behav Brain Funct..

[55] Brookes KJ, Hawi Z, Kirley A, Barry E, Gill M, Kent L (2008). Association of the steroid sulfatase (STS) gene with attention deficit hyperactivity disorder. Am J Med Genet B Neuropsychiatr Genet..

