# Supplementary material for: Attention deficit hyperactivity disorder: genetic association study in a cohort of Spanish children
Source: Behav Brain Funct. 2016 Jan 8;12:2. doi: 10.1186/s12993-015-0084-6 (PMC4706690; doi:10.1186/s12993-015-0084-6)
Supplement: Supplementary file 1 — Additional file 1: Table S1. Significant results of logistic regression for single markers considering a nominal p value <0.05. [file 12993_2015_84_MOESM1_ESM.doc]

| **Table S1**. Significant results of logistic regression for single markers (nominal p-value <0.05) | | | | | | | | |
| --- | --- | --- | --- | --- | --- | --- | --- | --- |
| **All population** | | | | | | | | |
| **Gene** | **Variant** | **Model** | **Genotype** | **Controls N (%)** | **Cases N (%)** | **OR (95% CI)** | **P-value** | **P-value corrected** |
|  |  |  | A/A-T/T | 204 (60) | 147 (50.7) | 1 |  |  |
| ***SLC6A2*** | **rs28386840** | Overdominant | A/T | 136 (40) | 143 (49.3) | 1.45 (1.05-2.00) | 0.0240 | 0.1118 |
|  |  |  | G/G | 281 (83.4) | 260 (90.3) | 1 |  |  |
| **SLC6A3** | **rs11564750** | Dominant | G/C-C/C | 56 (16.6) | 28 (9.7) | 0.53 (0.32-0.87) | 0.0096 | 0.0816 |
|  |  |  | -/- -/7 | 325 (96.2) | 287 (99.3) | 1 |  |  |
| ***DRD4*** | **Exon3 VNTR** | Recessive | 7/7 | 13 (3.8) | 2 (0.7) | 0.17 ( 0.04-0.78) | 0.0063 | 0.0714 |
|  |  |  | L/L-L/S | 241 (71.1) | 239 (83.3) | 1 |  |  |
| ***SLC6A4*** | **Promoter VNTR** | Recessive | S/S | 98 (28.9) | 48 (16.7) | 0.52 (0.35-0.77) | 0.0009 | **0.0153** |
|  |  |  | A/A | 122 (36) | 133 (45.9) | 1 |  |  |
| ***SNAP25*** | **rs3746544** | Dominant | A/C-C/C | 217 (64) | 157 (54.1) | 0.68 (0.49-0.94) | 0.0190 | 0.1077 |
|  |  |  | A/A-G/G | 177 (52.1) | 185 (63.8) | 1 |  |  |
| ***LPHN3*** | **rs2305339** | Overdominant | A/G | 163 (47.9) | 105 (36.2) | 0.57 (0.41-0.79) | 0.0007 | **0.0153** |
|  |  |  | A/A-G/G | 161 (47.4) | 169 (58.3) | 1 |  |  |
|  | **rs6551665** | Overdominant | A/G | 179 (52.6) | 121 (41.7) | 0.68 (0.49-0.94) | 0.0180 | 0.1077 |
|  |  |  | A/A-A/G | 274 (81.5) | 211 (73) | 1 |  |  |
|  | **rs6858066** | Recessive | G/G | 62 (18.4) | 78 (27) | 1.55 (1.05-2.28) | 0.0260 | 0.118 |
| **Combined subtype** | |  |  |  |  |  |  |  |
| **Gene** | **Variant** | **Model** | **Genotype** | **Controls N (%)** | **Cases N (%)** | **OR (95% CI)** | **P-value** | **P-value corrected** |
|  |  |  | A/A | 174 (51.2) | 65 (37.1) | 1 |  |  |
| ***SLC6A2*** | **rs28386840** | Dominant | A/T-T/T | 166 (48.8) | 110 (62.9) | 1.76 (1.19-2.59) | 0.0041 | **0.0318** |
|  |  |  | G/G | 281 (83.4) | 161 (92.5) | 1 |  |  |
|  |  |  | G/C | 51 (15.1) | 13 (7.5) | 0.40 (0.21-0.77) | 0.0026 | **0.0269** |
| ***SLC6A3*** | **rs11564750** | Log-additive | C/C | 5 (1.5) | 0 (0) |  |  |  |
|  |  |  | G/G | 203 (59.7) | 119 (68) | 1 |  |  |
| ***DDC*** | **rs6592961** | Dominant | G/A-A/A | 137 (40.3) | 56 (32) | 0.64 (0.43-0.95) | 0.0260 | 0.1624 |
|  |  |  | L/L-L/S | 241 (71.1) | 151 (87.3) | 1 |  |  |
| ***SLC6A4*** | **Promoter VNTR** | Recessive | S/S | 98 (28.9) | 22 (12.7) | 0.37 (0.22-0.62) | 0.0001 | **0.0031** |
|  |  |  | A/A-G/G | 177 (52.1) | 114 (65.1) | 1 |  |  |
| ***LPHN3*** | **rs2305339** | Overdominant | A/G | 163 (47.9) | 61 (34.9) | 0.51 (0.34-0.76) | 0.0008 | **0.0124** |
| **Inattentive subtype** | |  |  |  |  |  |  |  |
| **Gene** | **Variant** | **Model** | **Genotype** | **Controls N (%)** | **Cases N (%)** | **OR (95% CI)** | **P-value** | **P-value corrected** |
|  |  |  | L/L-L/S | 321 (94.4) | 88 (86.3) | 1 |  |  |
| ***SLC6A3*** | **Intron8 VNTR** | Recessive | S/S | 19 (5.6) | 14 (13.7) | 2.63 (1.26-5.47) | 0.0120 | 0.1318 |
|  |  |  | -/- | 238 (70.4) | 68 (66.7) | 1 |  |  |
| ***DRD4*** | **Exon3 VNTR** | Codominant | -/7 | 87 (25.7) | 34 (33.3) | 1.36 (0.84-2.20) | 0.0150 | 0.1318 |
|  |  |  | A/A | 122 (36) | 49 (48) | 1 |  |  |
| ***SNAP25*** | **rs3746544** | Dominant | A/C-C/C | 217 (64) | 53 (52) | 0.63 (0.40-0.98) | 0.0430 | 0.2077 |
|  |  |  | G/G | 193 (56.8) | 48 (47.1) | 1 |  |  |
|  |  |  | G/A | 126 (37.1) | 42 (41.2) | 1.44 (1.02-2.03) | 0.0350 | 0.2077 |
| ***LPHN3*** | **rs1397548** | Log-additive | A/A | 21 (6.2) | 12 (11.8) |  |  |  |
|  |  |  | A/A | 112 (32.9) | 47 (46.1) | 1 |  |  |
|  | **rs6551665** | Dominant | A/G-G/G | 228 (67.1) | 55 (53.9) | 0.58 (0.37-0.90) | 0.0170 | 0.1318 |
|  |  |  | A/A-G/G | 274 (81.5%) | 71 (70.3) | 1 |  |  |
|  | **rs6858066** | Recessive | G/G | 62 (18.4%) | 30 (29.7) | 1.89 (1.13-3.15) | 0.0160 | 0.1318 |
| **Female** |  |  |  |  |  |  |  |  |
| **Gene** | **Variant** | **Model** | **Genotype** | **Controls N (%)** | **Cases N (%)** | **OR (95% CI)** | **P-value** | **P-value corrected** |
|  |  |  | A/A-A/T | 101 (87.8) | 56 (96.5) | 1 |  |  |
| ***SLC6A2*** | **rs28386840** | Recessive | T/T | 14 (12.2) | 2 (3.5) | 0.24 (0.04 - 0.90) | 0.0335 | 0.1553 |
|  |  |  | A/A-A/G | 90 (79.7) | 53 (91.4) | 1 |  |  |
| ***SLC6A3*** | **rs2550948** | Recessive | G/G | 23 (20.4) | 5 (8.6) | 0.31(0.10 - 0.83) | 0.0189 | 0.1435 |
|  |  |  | C/C-C/T | 94 (81.7) | 53 (91.4) | 1 |  |  |
|  | **rs2652511** | Recessive | T/T | 21 (18.3) | 5 (8.6) | 0.34(0.11 - 0.92) | 0.0333 | 0.1553 |
|  |  |  | G/G | 96 (83.5) | 56 (96.5) | 1 |  |  |
|  |  |  | G/C | 14 (12.2) | 2 (3.5) | 0.22 (0.04 - 0.70) | 0.0061 | 0.1099 |
|  | **rs11564750** | Log-additive | C/C | 5 (4.3) | 0 |  |  |  |
|  |  |  | -/- | 83 (72.8) | 35 (60.3) | 1 |  |  |
| ***DRD4*** | **Exon3 VNTR** | Codominant | -/7 | 26 (22.8) | 23 (39.7) | 2.12(1.06-4.25) | 0.0110 | 0.1435 |
|  |  |  | S/S | 41 (35.6) | 12 (20.7) | 1 |  |  |
|  |  |  | S/L | 52 (45.2) | 28 (48.3) | 0.62(0.39 - 0.97) | 0.0374 | 0.1553 |
| ***SLC6A4*** | **Promoter VNTR** | Log-additive | L/L | 22 (19.1) | 18 (31) |  |  |  |
|  |  |  | A/A | 34 (296) | 28 (48.3) | 1 |  |  |
|  |  |  | A/C | 64 (55.6) | 27 (46.5) | 0.5(0.29 - 0.84) | 0.0084 | 0.1099 |
| ***SNAP25*** | **rs3746544** | Log-additive | C/C | 17 (14.8) | 3 (5.2) |  |  |  |
|  |  |  | A/A-A/G | 107 (93) | 46 (79.3) | 1 |  |  |
| ***LPHN3*** | **rs6551665** | Recessive | G/G | 8 (7) | 12 (20.7) | 3.53(1.36 - 9.66) | 0.0097 | 0.1099 |
|  |  |  | T/T | 54 (47) | 34 (59.6) | 1 |  |  |
|  |  |  | T/A | 47 (40.9) | 21 (36.8) | 0.58 (0.34-0.99) | 0.0411 | 0.1553 |
|  | **rs1868790** | Log-additive | A/A | 14 (12.2) | 2 (3.5) |  |  |  |
| **Male** |  |  |  |  |  |  |  |  |
| **Gene** | **Variant** | **Model** | **Genotype** | **Controls N (%)** | **Cases N (%)** | **OR (95% CI)** | **P-value** | **P-value corrected** |
|  |  |  | A/A | 120 (53.3) | 94 (40.5) | 1 |  |  |
| ***SLC6A2*** | **rs28386840** | Dominant | A/T-T/T | 105 (46.7) | 138 (59.5) | 1.67(1.15 - 2.42) | 0.0067 | 0.0703 |
|  |  |  | G/G | 185 (83.3) | 204 (88.7) | 1 |  |  |
| ***SLC6A3*** | **rs11564750** | Codominant | G/C | 37 (16.7) | 24 (10.4) | 0.59(0.34 - 1.02) | 0.0451 | 0.1677 |
|  |  |  | L/L-L/S | 214 (95.1) | 210 (90.5) | 1 |  |  |
|  | **Intron8 VNTR** | Recessive | S/S | 11 (4.9) | 22 (9.5) | 2.16(1.04 - 4.76) | 0.0397 | 0.1677 |
|  |  |  | -/- -/7 | 216 (96.4) | 229 (99.1) | 1 |  |  |
| ***DRD4*** | **Exon3 VNTR** | Recessive | 7/7 | 8 (3.6) | 2 (0.9) | 0.23(0.03-0.95) | 0.0415 | 0.1677 |
|  |  |  | L/L-L/S | 167 (74.5) | 193 (84.3) | 1 |  |  |
| ***SLC6A4*** | **Promoter VNTR** | Recessive | S/S | 57 (25.4) | 36 (15.7) | 0.53(0.33 - 0.84) | 0.0068 | 0.0703 |
|  |  |  | G/G-G/A | 214 (95.5) | 207 (90.8) | 1 |  |  |
| ***GRM7*** | **rs3792452** | Recessive | A/A | 10 (4.5) | 21 (9.2) | 2.14(1.00 - 4.86) | 0.0487 | 0.1677 |
|  |  |  | G/G | 55 (24.4) | 77 (33.3) | 1 |  |  |
| ***CDH13*** | **rs6565113** | Dominant | G/T-T/T | 170 (75.6) | 154 (66.7) | 0.64(0.42 - 0.96) | 0.0298 | 0.1677 |
|  |  |  | G/G-G/A | 213 (94.7) | 206 (89.2) | 1 |  |  |
| ***LPHN3*** | **rs1397548** | Recessive | A/A | 12 (5.3) | 25 (10.8) | 2.15 (1.07 - 4.54) | 0.0315 | 0.1677 |
|  |  |  | A/A | 83 (36.9) | 128 (55.2) | 1 |  |  |
|  | **rs2305339** | Codominant | A/G | 128 (56.9) | 81 (34.9) | 0.41 (0.28 - 0.60) | 0.0000 | **0.0001** |
| OR, odds ratio; CI, *confidence interval*  P-values corrected based on the Benjamini&Hochberg method | | | | | | | | |
